# Association between Biomarkers of Inflammation and 10-Year Changes in Aortic Stiffness: The Multi-Ethnic Study of Atherosclerosis

**DOI:** 10.3390/jcm12155062

**Published:** 2023-08-01

**Authors:** Rithvik Swamynathan, Vinithra Varadarajan, Hieu Nguyen, Colin O. Wu, Kiang Liu, David A. Bluemke, Nadjia Kachenoura, Alban Redheuil, João A. C. Lima, Bharath Ambale-Venkatesh

**Affiliations:** 1 Department of Cardiology, Johns Hopkins University School of Medicine, Baltimore, MD 21287, USA; 2 National Institutes of Health, Bethesda, MD 20892, USA; 3 Department of Preventive Medicine, Northwestern University Medical School, Chicago, IL 60622, USA; 4 Department of Radiology, University of Wisconsin School of Medicine and Public Health, Madison, WI 53705, USA; 5 CNRS, INSERM, Laboratoire d’Imagerie Biomédicale, LIB, Sorbonne Université, 75006 Paris, Francealban.redheuil@gmail.com (A.R.); 6 Imagerie Cardiovasculaire et Thoracique, Institut de Cardiologie, Groupe Hospitalier Pitié Salpêtrière APHP, 75013 Paris, France; 7 Department of Radiology, Johns Hopkins University School of Medicine, Baltimore, MD 21287, USA

**Keywords:** aorta, distensibility, pulse wave velocity, inflammation, MRI

## Abstract

*Background*. Chronic inflammation is associated with incident cardiovascular events. We study the association between biomarkers of inflammation and subclinical vascular dysfunction measured as proximal aortic stiffness. *Methods.* MRI imaging was performed in the Multi-Ethnic Study of Atherosclerosis (MESA) at baseline (2000) and at the 10-year follow-up. Aortic arch pulse wave velocity (PWV) and ascending and descending aorta distensibility (AAD, DAD) were measured in 1223 asymptomatic individuals at both exams. Linear regression was used to study the association of baseline inflammation—C-reactive protein (CRP), interleukin-6 (IL6), and fibrinogen (Fib)—with baseline and 10-year changes in aortic stiffness (PWV, AAD, DAD). *Results*. The mean age of the participants was 59 ± 9 years, 47.8% of them were men, 32.6% were hypertensive at baseline, and 7.6% were diabetic. At baseline and follow-up, the mean AAD values were, respectively, 1.73 × 10^−3^ mmHg^−1^ and 1.57 × 10^−3^ mmHg^−1^, the mean DAD values were 2.19 × 10^−3^ mmHg^−1^ and 1.99 × 10^−3^ mmHg^−1^, and the mean PWV values were 8.10 m/s and 8.99 m/s. At baseline, the AAD (in 10^−3^ mmHg^−1^) and DAD (in 10^−3^ mmHg^−1^) were inversely associated with CRP (in mg/L) (AAD coeff: −0.047, *p*-value: 0.011, DAD coeff: −0.068, *p*-value: <0.001) and IL6 (in pg/mL) (AAD coeff: −0.098, *p*-value: 0.003, DAD coeff: −0.14, *p*-value: <0.001) in a univariable analysis but not after adjustment for demographic variables or cardiovascular risk factors. The baseline DAD was inversely associated with Fib (in mg/dL) (coeff: −0.334, *p*-value: 0.001). The baseline PWV (in m/s) was positively associated with IL6 (in pg/mL) in a univariable analysis (coeff: 0.054, *p*-value: 0.014). In a longitudinal analysis, the 10-year changes in DAD were inversely associated with CRP, even after adjustment for demographics and risk factors (DAD coeff: −0.08, *p*-value 0.044). *Conclusions.* Higher CRP levels at baseline were independently associated with a 10-year increase in aortic stiffness, measured as decreased aortic distensibility.

## 1. Introduction

Inflammation has been linked to incident cardiovascular disease and an increased risk of heart failure. Similarly, aortic stiffness has been linked to changes in the aortic structure and remodeling, which can place individuals at an increased risk for cardiovascular disease. Inflammatory biomarkers analyses from circulating blood samples are relatively non-invasive procedures. These may be used to monitor inflammatory pathways and understand the magnitude of the inflammatory response at a given time point. Such monitoring can be performed by quantitatively measuring inflammatory biomarkers, such as interleukin-6, C-reactive protein, and fibrinogen [1]. Furthermore, these biomarkers have been linked to cardiovascular fibrosis [2]. The loss of elastin and the subsequent replacement by collagen in the aortic wall with aging may be accelerated in the presence of chronic or repeated inflammation [3]. Multiple studies document that vascular stiffness is associated with inflammation [4] Higher levels of various inflammatory markers are associated with a risk of future atherosclerotic vascular diseases. These systemic markers include C-reactive protein (CRP), proinflammatory cytokines, and soluble adhesion molecules. Among these markers, CRP and interleukin-6 (IL-6), in particular, were associated with vascular events, independent of vascular risk factors [5,6,7].

The differential elasticity of arteries helps regulate the cardiovascular system, including monitoring blood pressure and cyclical expansion and collapse based on the cardiac contraction cycle [8]. Reduced proximal aortic elasticity limits the ability of the aorta to adjust to changes in luminal pressures and to properly regulate blood flow, and as a result, the risk of CVD increases as the stiffness increases and the aortic structure changes. MRI is a reference method for non-invasively assessing proximal aortic stiffness. 

Prior studies have shown a direct relationship between inflammatory biomarkers and arterial stiffness in patients with and without cardiovascular disease [1,4,9]. This study will conduct the analysis in a larger, multiethnic, and initially asymptomatic population. Furthermore, while prior studies have established the cross-sectional relationships between inflammation and aortic stiffness, we hope to achieve a better understanding of the strength of the longitudinal associations. The Multi-Ethnic Study of Atherosclerosis (MESA) database was used in this work, as the cohort specialized in studying a large, diverse population of asymptomatic participants to understand the progression of cardiovascular disease. Evaluating the relationship between inflammation and aortic stiffness at baseline and during its longitudinal change can be clinically relevant, giving the physicians more insight into the risks of cardiovascular disease. A longitudinal approach can fill gaps in understanding the relationship between inflammation and aortic stiffness by eliminating cross-sectional bias. 

## 2. Methods

### 2.1. Population

**Figure 1:**
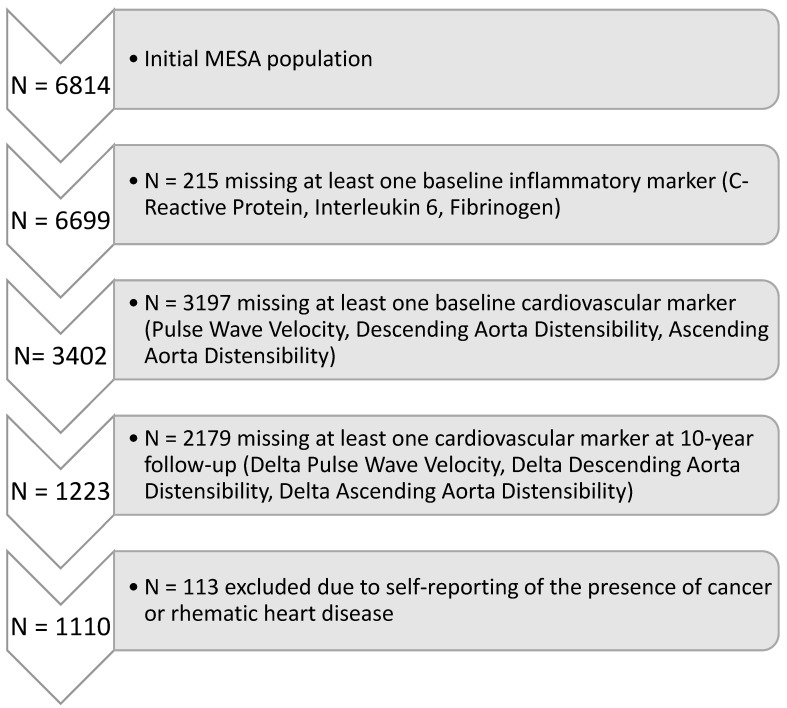
Flowchart of Participant Selection.

The Multi-Ethnic Study of Atherosclerosis is a longitudinal study evaluating 6814 individuals free of clinical cardiovascular disease at the baseline enrollment in 2000. The present analysis included only patients in whom all three biomarkers of inflammation (IL-6; CRP; fibrinogen) were available at baseline and all measures of arterial stiffness (pulse wave velocity, ascending aortic distensibility, descending aortic distensibility) were available at baseline and at the 10-year follow-up. Of the initial 6814, 215 individuals were missing at least one biomarker of inflammation (Interleukin-6, C-reactive protein, Fibrinogen). Of the remaining 6599, 3197 individuals were missing at least one measure of arterial stiffness (pulse wave velocity, ascending aortic distensibility, descending aortic distensibility), and 2179 more individuals were missing at least one measure of arterial stiffness at the 10-year follow-up. Aortic MRI was not performed on a subset of individuals, resulting in the missing values. Finally, 113 more individuals were excluded due to conditions that may impact the level of inflammation (cancer or rheumatic heart disease), resulting in the sample size of 1110 for both baseline and longitudinal (10-year follow-up) analyses. This study was approved by all six participating MESA institutions and by the IRB at Johns Hopkins University Cardiovascular Imaging Core Laboratory. The participants of the study gave informed consent. MESA also gave consent for publication. The detailed flowchart for participant selection is shown in Figure 1.

### 2.2. Magnetic Resonance Imaging

MRI was performed using 1.5 T whole-body MRI systems at both the MESA baseline exam and the 10-year follow-up. Phase contrast cine gradient echo MRI with electrocardiographic gating was performed to evaluate the aortic flow and distensibility of the aorta. Images of the ascending and descending aorta were obtained in the transverse plane at the level of the right pulmonary artery perpendicular to the aortic vessel lumen. The typical imaging parameters include: a repetition time of 5.8 ms, an echo time of 3.5 ms, a matrix size of 128 × 128 pixels, and a spatial resolution of 1.2 × 1.2 × 8 mm [3,10].

**Figure 2:**
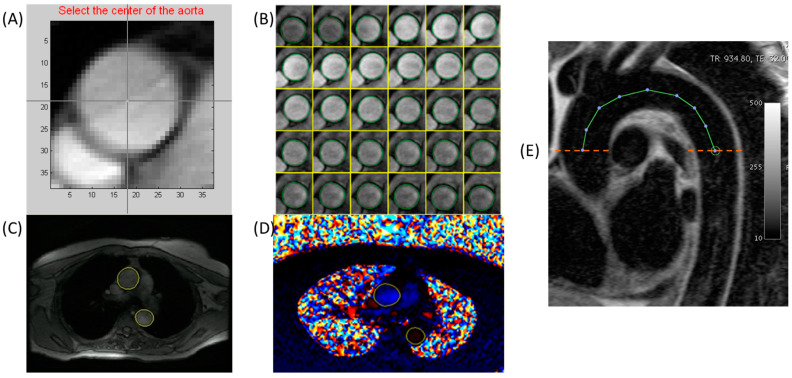
Methods for aortic segmentation and parameter estimation illustrated. Top row shows semi-automated segmentation of the aorta using ARTFUN. (**A**) Identification of the center of the aorta, (**B**) Semi-automated segmentation, (**C**,**D**) Final semi-automated segmentation of the ascending and descending aortic cross-sections in the magnitude and phase images, respectively. (**E**) Aortic arch length was calculated from candy-cane images. Pulse wave velocity was calculated using arch length and the flow from the corresponding phase-contrast images in the ascending and descending arms of the aorta.

To determine aortic distensibility, the minimum and maximum cross-sectional areas of the ascending and descending aorta were measured from the modulus images with an automated time-resolved contour detection software (ARTFUN v3 INSERM U1146), as previously described by Herment et al. [11,12] and used by Redheuil et al. [13]. Pointing to the center of the ascending and descending aorta on the mean intensity projection image of each dataset is needed for this phantom-validated segmentation method. Additional manual intervention for correcting the semi-automatically generated segmentation was performed, as necessary. A detailed illustration of the segmentation process is shown in Figure 2.

Distensibility, describing the ability of a vessel to stretch, was then calculated as:

AAD (mmHg^−1^) = [ascending aorta maximal lumen area (cm^2^)—ascending aorta minimal lumen area (cm^2^)]/[PP (mmHg) x ascending aorta minimal lumen area (cm^2^)].

DAD (mmHg^−1^) = [descending aorta maximal lumen area (cm^2^)—descending aorta minimal lumen area (cm^2^)]/[PP (mmHg) x descending aorta minimal lumen area (cm^2^)], where PP is the brachial pulse pressure calculated from the average systolic and diastolic pressures measured before and after MRI in mmHg.

Applying modulus time-resolved contours to the velocity images, ARTFUN software allowed us to obtain the transit-time between the ascending and descending aorta, calculated as the time-shift between their respective flow-rate curves. Aortic sagittal oblique planes with a black-blood sequence were also acquired to enable the measurement of the distance between the ascending and descending aorta. PWV, describing the velocity of blood pulse pressure, was then calculated as: 

PWV (m/s) = ascending to descending aorta distance (m)/ascending to descending aorta transit time (ms).

### 2.3. Inflammatory Biomarker Measurement

Three biomarkers were selected for use in this study. These include C-reactive Protein (CRP), Interleukin-6 (IL-6), and Fibrinogen. When evaluating biomarkers, two characteristics were prioritized: a high availability of data, and the impact of the biomarker in the inflammatory process. CRP, IL-6, and Fibrinogen data were available for a majority of the participants. Only 215 individuals of the MESA population (n = 6814) were missing all three biomarkers. The biomarkers were evaluated at the University of Vermont [14] from blood samples of participants who avoided exercise, smoking, and eating for 12 h prior to collection. Fibrinogen was measured through a BN^TM^II nephelometer [15]. CRP and IL-6 were measured in blood samples at baseline using a protocol used in the Cardiovascular Health Study by Cushman et al. [16]. 

### 2.4. Statistical Analysis

Kolmogorov–Smirnov tests were performed initially, and the measures were logarithmic-transformed, as appropriate. The logarithms of each biomarker of inflammation and the logarithms of each vascular stiffness marker all passed the Kolmogorov–Smirnov test for normality. Further, linear regression models were used to associate inflammation with aortic stiffness. Correlation coefficients were calculated. Three models were used: a univariate model and two multivariable models. The univariate model was simply the association of the inflammatory markers with aortic stiffness markers, with no adjustments. The first multivariable model adjusted for the demographic variables: age, sex, and race. The second multivariable model adjusted for age, sex, race, body mass index (BMI), diabetes mellitus classification, hypertension, high-density lipoprotein, smoking status (measured in pack years), and lipid-lowering medication use. This analysis was conducted both cross-sectionally and longitudinally. For the longitudinal analysis, changes in aortic stiffness markers were calculated by subtracting baseline values from 10-year follow-up values. 

## 3. Results

Figure 1 identifies the cohorts of the participants in this study, which included 1110 participants from the total sample of 6814. At baseline, the mean age of the population was 62 ± 10 years, with the selected sample mean age of 59 ± 9 years. A total of 47.2% of the study population were male, and 47.8% of the selected sample were male. Participant characteristics are shown in Table 1. In brief, the selected sample was 40.4% Caucasian, 21.6% African-American, 28.5% Chinese, and 9.5% Hispanic. The participant mean BMI at baseline was 27.5 ± 5.0 kg/m^2^. A total of 32.6% were hypertensive at baseline. The percentage of individuals with treated/untreated diabetes was 7.6% at baseline. The percentage of current smokers was 10.8% at baseline. The LDL cholesterol level was 117.0 ± 30 mg/dL at baseline. The HDL cholesterol level was 51.2 ± 14.8 mg/dL at baseline. There were no statistically significant differences between the MESA population and the study population.

The mean biomarker values for the selected sample were for IL6 1.35 ± 1.04 pg/mL, for CRP 3.33 ± 5.26 mg/L, and for Fib 332.4 ± 66.7 mg/dL. The vascular stiffness markers were measured at baseline and year 10. At baseline, the mean pulse wave velocity was 8.10 ± 4.94 m/s, the mean ascending aortic distensibility was 1.73 ± 1.35 × 10^−3^ mmHg^−1^, and the mean descending aortic distensibility was 2.19 ± 1.52 × 10^−3^ mmHg^−1^. At the ten-year follow-up, the mean pulse wave velocity was 8.99 ± 4.22 m/s. The mean ascending aortic distensibility was 1.57 ± 1.10 × 10^−3^ mmHg^−1^, and the mean descending aortic distensibility was 1.99 ± 1.32 × 10^−3^ mmHg^−1^. Table 1 describes the characteristics of the population in both the complete MESA sample (n = 6814) and the selected sample (n = 1110). There were no statistically significant differences between the MESA population and the study population. 

Cross-sectionally, at baseline, each biomarker of inflammation studied was significantly and negatively associated with descending aorta distensibility (in 10^−3^ mmHg^−1^) (CRP (in mg/L) coeff: −0.068, *p*-val: <0.001; IL-6 (in pg/mL) coeff: −0.142, *p*-val: <0.001; Fib (in mg/dL) coeff: −0.334, *p*-val: 0.001). CRP and IL6 are negatively associated with ascending aorta distensibility (in 10^−3^ mmHg^−1^) (CRP (in mg/L) coeff: −0.047, *p*-val: 0.011; IL-6 (in pg/mL) coeff: −0.098, *p*-val: 0.003) in the univariate model, i.e., greater inflammation levels were associated with lower AAD and DAD values and, hence, increased stiffness. However, such associations were not independent of demographics and risk factors. Age was a significant confounder for the association between AAD and inflammation markers (adjustments for age alone rendered the associations not significant). For the associations with DAD and IL-6, the addition of age and race confounded the association. Age was inversely associated with distensibility, while PWV was directly associated with age. Gender was not significantly associated with any of the stiffness parameters. African-Americans had reduced AAD and DAD compared to Caucasians. Chinese-Americans had a higher AAD compared to Caucasians, while Hispanics had a higher PWV compared to Caucasians. Furthermore, PWV (in m/s) was positively associated with IL6 (in pg/mL) in the cross-sectional analysis at baseline (coeff: 0.054, *p*-val: 0.014) in the univariate model. Table 2 shows the coefficients and *p*-values of every cross-sectional association performed. 

Longitudinally, higher levels of CRP (in mg/L) at baseline trended towards an association with larger decreases in AAD (in 10^−3^ mmHg^−1^) (coeff: −0.05, *p*-val: 0.07) and were associated with larger decreases in DAD (in 10^−3^ mmHg^−1^) (coeff:−0.08, *p*-val: 0.028) over the 10 years of follow-up after an adjustment for risk factors and demographics. Finally, IL-6 (in pg/mL) was positively associated with delta PWV (m/s) in the univariate model (coeff: 0.040, *p*-val: 0.019). Table 3 shows the coefficients and *p*-values of the longitudinal analysis performed using baseline traditional risk factor values. Additional adjustments to model three with aspirin use, NSAID use, and ACE use did not modify the associations, and none of them were significantly associated with the change in stiffness over 10 years.

## 4. Discussion

In a large multi-ethnic population, aortic distensibility and the biomarkers of inflammation were negatively associated cross-sectionally. Cross-sectional associations between inflammatory biomarkers and aortic stiffness are largely influenced by demographic variables and traditional risk factors, showing that further research can help determine risk factors that confound the relationship between inflammation and aortic stiffness. Over a 10-year follow-up period, decreased aortic distensibility over 10 years was independently associated with higher levels of CRP at baseline. Inflammation may play a key role in the long-term decrease in distensibility and increase in aortic stiffness in middle-to-older-aged individuals initially free of overt CV disease. 

The development of diagnostic biomarkers of acute cardiovascular disease remains an important topic of interest given the potential use to aid in early diagnosis. Cardiac biomarkers of ischemia and heart failure have already been proven to be clinically useful. Biomarkers of aortic diseases are also needed, especially for life-threatening conditions such as aortic dissection. Prior studies within the MESA database and in other populations have shown the association of the aortic arch pulse wave velocity and proximal aortic distensibility as markers of incident events—including atherosclerotic cardiovascular disease as well as mortality. While hypertension and aging are established factors contributing to arterial stiffness, the role of inflammation in the stiffening of the arteries is less well understood. Several studies have assessed the association between PWV and distensibility with various inflammatory markers [17]. However, the effect of inflammation on longitudinal changes in aortic stiffness has been studied far less, particularly in those without primary inflammatory diseases. The Whitehall study found the baseline CRP, IL-6, and fibrinogen to be significantly associated with the cfPWV measured 10 years later [18]. Similarly, the Caerphilly study demonstrated a significant relationship between the baseline CRP values and carotid femoral PWV (cfPWV) measured 20 years later [19]. Another study showed that changes in vascular distensibility were associated with levels of CRP [20]. However, other prospective studies over shorter follow-up periods did not find inflammatory markers at baseline to be associated with the rate of change of stiffness measures [21,22]. This highlights the need for further prospective studies to understand the role of inflammation in the rate of change of vascular stiffness, particularly central arterial stiffness.

In 2011, Arnett et al. used the MESA database to evaluate the association between inflammation and left ventricular mass [1]. This study tested 12 biomarkers of inflammation and measured the fibrotic development of left ventricular mass. There was an association noted between CRP and left ventricular mass. In 2017, Mozos et al. tested the association of biomarkers of inflammation and cardiovascular disease outcomes [4]. They also noted an association between CRP and aortic stiffness indicators. Factors such as weight play a role in the association, showing that the association between aortic stiffness and inflammation is not independent. The association is confounded by other cardiovascular risk factors and becomes increasingly confounded at older ages. In a study conducted by Jabati et al., a positive relationship between inflammation and collagen turnover was observed, showing that increases in inflammation were linked to increased collagen turnover [23]. This study provides one indication of the pathways through which inflammation affects aortic stiffness, i.e., through increased collagen turnover. However, the relationship between inflammation and aortic stiffness may be more complex, and further biomarker studies are needed to elucidate these associations [17].

The results of the study showed the longitudinal association between aortic distensibility and CRP in all three models. However, this association was not seen between aortic distensibility and IL-6. IL-6 is a pro-inflammatory cytokine that plays a role in the inflammatory response that is upstream from CRP. IL-6 is a primary cytokine that plays a role in hepatic CRP production [24]. As an upstream cytokine, a direct association between IL-6 and aortic stiffness is more confounded than CRP by other pathways that affect aortic stiffness. Furthermore, it is likely that CRP is a downstream molecule that can be activated by other inflammatory pathways that play a more direct role in the vascular stiffening process. Finally, CRP has a longer half-life when compared to IL-6, meaning that it can be a more reliable and applicable metric to associate with vascular stiffness longitudinally [25]. A study by Sesso et al. similarly found an association between CRP and an increased risk of hypertension, while the association between IL-6 and the risk of hypertension was relatively weak [26]. 

Theoretically, inflammation increases aortic stiffness through the repetitive activation of the inflammatory pathway. As repetitive inflammatory events occur, the tissue is healed and replaced with fibrotic tissue. This tissue is less elastic, restricting blood flow and increasing arterial stiffness gradually. The results from this study support this theory. This suggests that taking anti-inflammatory medication may be suggested to slow down the aortic stiffening process. A study by Ridker et al. showed that targeting the interleukin-1B innate immunity pathway with anti-inflammatory medication led to a lower rate of cardiovascular events recurrence, when compared to the placebo [27]. However, there are multiple additional factors that can affect such an association. Certain traditional risk factors are more likely to cause increased inflammatory events. For example, obesity and age both result in more inflammatory events while also increasing arterial stiffness through other mechanisms. A further brief inspection of the role of aging in the relationship between stiffness and inflammation showed that adjustment for aging decreased the strength of the association between inflammation and aortic stiffness. It is likely that several factors can impact aortic stiffness at older age ranges, while at younger age ranges, higher levels of aortic stiffness can be attributed to increased inflammation more directly [28,29,30].

This study has a number of limitations. Testing for association using three vascular stiffness markers and three biomarkers can be very limiting, as was seen in this situation, as an association was not seen between any vascular markers and fibrinogen or between any inflammatory markers and PWV. Further, the study was conducted among individuals between the ages of 45 and 84. With an overall population mean age of 62 ± 10 years, this study did not evaluate the relationship between inflammation and aortic stiffness among younger populations. The availability of inflammatory marker data at baseline was a limitation, as individuals with abnormally high inflammatory marker concentrations due to cancer or other major diseases can skew the association. The adjustment for individuals without cancer or rheumatic heart disease allows for more accurate results. However, we did not have robust follow-up data on malignancies and other non-CVD events that may affect chronic inflammation during the follow-up period. This study included a multi-ethnic population. In our analysis, there was no significant (*p* < 0.05) interaction by race for the association between markers of inflammation and aortic stiffness for both the cross-sectional and longitudinal analysis. This may be because the effect sizes may not be large enough to be picked up within the given sample size.

In summary, the study of the association of biomarkers of inflammation and aortic stiffness showed that higher levels of baseline inflammatory biomarker levels were not associated with increased aortic stiffness cross-sectionally. However, in the longitudinal analysis, higher baseline CRP levels were independently associated with a 10-year increase in aortic stiffness.

## Figures and Tables

**Table 1:** Population Characteristics.

	Full Sample	Selected Sample (Year 1)	Selected Sample (Year 10)
N	6814	1110 (16.2)	1098 (16.1)
Age (years)	62 ± 10	59 ± 9	69 ± 9
Male	3213 (47.2)	531 (47.8)	531 (47.8)
Race:			
Caucasian	2622 (38.5)	448 (40.4)	448 (40.4)
Chinese	804 (11.8)	240 (21.6)	240 (21.6)
Black	1892 (27.8)	316 (28.5)	316 (28.5)
Hispanic	1496 (21.9)	106 (9.5)	106 (9.5)
Body Mass Index (kg/m^2^)	28.3 ± 5.5	27.5 ± 5.0	28.5 ± 5.7
Obese (BMI > 30 kg/m^2^)	2196 (32.2)	302 (27.2)	319 (28.7)
Systolic Blood Pressure (mmHg)	126.6 ± 21.5	132.6 ± 20.0	126.1 ± 16.8
Diastolic Blood Pressure (mmHg)	71.9 ± 10.3	77.5 ± 11.0	71.4 ± 11.7
Pulse Pressure (mmHg)	54.7 ± 31.8	55.1 ± 31.0	54.7 ± 28.5
Antihypertensive medication	2536 (37.2)	362 (32.6)	553 (55.4)
Controlled Hypertension	1547 (22.7)	234 (21.1)	445 (40.1)
Low-Density Lipoprotein cholesterol (mg/dL)	117.2 ± 31.5	117.0 ± 30	105.4 ± 32.6
High-Density Lipoprotein cholesterol (mg/dL)	50.1 ± 14.8	51.2 ± 14.8	56.0 ± 16.8
Lipid-lowering Medication	1105 (16.3)	172 (15.5)	428 (39.0)
Smoking status			
Never	3418 (50.3)	646 (58.5)	576 (52.5)
Former	2487 (36.6)	340 (30.7)	436 (39.7)
Current	887 (13.1)	119 (10.8)	86 (7.8)
Diabetes	859 (12.6)	95 (7.6)	220 (20.0)
Interleukin-6 (pg/mL)	1.56 ± 1.23	1.35 ± 1.04	xx
C-Reactive Protein (mg/L)	3.78 ± 5.89	3.33 ± 5.26	xx
Fibrinogen (mg/dL)	346.8 ± 74.0	332.4 ± 66.7	xx
Pulse Wave Velocity (m/s)		8.10 ± 4.94	8.99 ± 4.22
Ascending Aortic Distensibility (10^−3^ mmHg^−1^)		1.73 ± 1.35	1.57 ± 1.10
Descending Aortic Distensibility (10^−3^ mmHg^−1^)		2.19 ± 1.52	1.99 ± 1.32

Values in brackets represent overall percentage.

**Table 2:** Cross-Sectional Analysis at Baseline.

	Log PWV (m/s)			Log AAD (10^−3^ mmHg^−1^)			Log DAD (10^−3^ mmHg^−1^)		
	Model 1	Model 2	Model 3	Model 1	Model 2	Model 3	Model 1	Model 2	Model 3
Log CRP (mg/L)	0.022 (0.07)	0.014 (0.23)	0.011 (0.39)	**−0.047 (0.01)**	−0.020 (0.27)	−0.014 (0.47)	**−0.068 (<0.01)**	**−0.033 (0.04)**	−0.007 (0.68)
Log IL-6 (pg/mL)	**0.054 (0.01)**	−0.0023 (0.91)	−0.010 (0.66)	**−0.098 (<0.01)**	−0.003 (0.92)	0.016 (0.63)	**−0.142 (<0.01)**	−0.032 (0.25)	0.021 (0.49)
Log Fib (mg/dl)	0.13 (0.06)	−0.029 (0.67)	−0.025 (0.71)	−0.174 (0.10)	0.013 (0.20)	0.158 (0.13)	**−0.334 (<0.01)**	−0.025 (0.78)	0.080 (0.38)

Coefficient and *p*-value (in parentheses) for association of baseline inflammatory markers and baseline aortic distensibility. N = 1110. Statistically significant values (*p* < 0.05) are bolded. Model 1: univariate; Model 2: Demographic Variables: age, sex, race; Model 3: Demographic Variables + Traditional Risk Factors: age, sex, race, diabetes mellitus classification, hypertension, high-density lipoprotein, smoking status (measured in pack years), BMI, and lipid-lowering medication use; IL6—Interleukin 6; CRP—C-reactive protein; Fib—Fibrinogen; PWV—Pulse Wave Velocity; AAD—Ascending Aortic Distensibility; DAD—Descending Aortic Distensibility.

**Table 3:** Longitudinal Analysis.

	dPWV (m/s)			dAAD (10^−3^ mmHg^−1^)			dDAD (10^−3^ mmHg^−1^)		
	Model 1	Model 2	Model 3	Model 1	Model 2	Model 3	Model 1	Model 2	Model 3
Log CRP (mg/L)	−0.03 (0.78)	−0.05 (0.67)	−0.08 (0.55)	**−0.05 (0.07)**	**−0.05 (0.09)**	**−0.06 (0.08)**	**−0.08 (0.028)**	**−0.09 (0.013)**	**−0.08 (0.044)**
Log IL-6 (pg/mL)	**0.040 (0.04)**	0.06 (0.76)	0.08 (0.73)	**−0.10 (0.07)**	−0.06 (0.25)	−0.08 (0.20)	**−0.09 (0.16)**	−0.07 (0.25)	−0.05 (0.50)
Log Fib (mg/dl)	−0.34 (0.61)	**−1.47 (0.03)**	−1.27 (0.06)	−0.15 (0.39)	0.01 (0.97)	−0.04 (0.84)	−0.05 (0.77)	−0.01 (0.97)	0.18 (0.62)

Coefficient and *p*-value (in parentheses) for the association of baseline inflammatory markers and 10-year change in aortic distensibility. N = 1110. Statistically significant values are bolded. Model 1: univariate + baseline MRI variable; Model 2: Demographic Variables: age, sex, race; Model 3: Demographic Variables + Traditional Risk Factors: age, sex, race, diabetes mellitus classification, hypertension, high-density lipoprotein, smoking status (measured in pack years), BMI, and lipid-lowering medication use. IL6—Interleukin 6; CRP—C-reactive protein; Fib—Fibrinogen; PWV—Pulse Wave Velocity; AAD—Ascending Aortic Distensibility; DAD—Descending Aortic Distensibility.

## Data Availability

Data is available through the MESA steering committee to all interested investigators.

## Abbreviations

AAD	Ascending Aortic Distensibility
ARTFUN	Arterial Function Software
BMI	Body Mass Index
CRP	C-reactive protein
CVD	Cardiovascular Disease
DAD	Descending Aortic Distensibility
Fib	Fibrinogen
HDL	High-density Lipoprotein
IL-6	Interleukin 6
IRB	Institutional Review Board
LDL	Low-density Lipoprotein
MESA	Multi-Ethnic Study of Atherosclerosis
MRI	Magnetic Resonance Imaging
PP	Pulse Pressure
PWV	Pulse Wave Velocity

## References

[1] Arnett D.K., McClelland R.L., Bank A., Bluemke D.A., Cushman M., Szalai A.J., Jain N., Gomes A.S., Heckbert S.R., Hundley W.G. (2011). Biomarkers of inflammation and hemostasis associated with left ventricular mass: The Multiethnic Study of Atherosclerosis (MESA). Int. J. Mol. Epidemiol. Genet..

[2] Marques M.D., Nauffal V., Ambale-Venkatesh B., Vasconcellos H.D., Wu C., Bahrami H., Tracy R.P., Cushman M., Bluemke D.A., Lima J.A.C. (2018). Association Between Inflammatory Markers and Myocardial Fibrosis. Hypertension.

[3] Ohyama Y., Ambale-Venkatesh B., Noda C., Kim J.-Y., Tanami Y., Teixido-Tura G., Chugh A.R., Redheuil A., Liu C.-Y., Wu C.O. (2017). Aortic Arch Pulse Wave Velocity Assessed by Magnetic Resonance Imaging as a Predictor of Incident Cardiovascular Events. Hypertension.

[4] Mozos I., Malainer C., Horbańczuk J., Gug C., Stoian D., Luca C.T., Atanasov A.G. (2017). Inflammatory Markers for Arterial Stiffness in Cardiovascular Diseases. Front. Immunol..

[5] Sattar N., Murray H.M., McConnachie A., Blauw G.J., Bollen E.L.E.M., Buckley B.M., Cobbe S.M., Ford I., Gaw A., Hyland M. (2007). C-Reactive Protein and Prediction of Coronary Heart Disease and Global Vascular Events in the Prospective Study of Pravastatin in the Elderly at Risk (PROSPER). Circulation.

[6] Suzuki T., Bossone E., Sawaki D., Jánosi R.A., Erbel R., Eagle K., Nagai R. (2013). Biomarkers of aortic diseases. Am. Heart J..

[7] McCabe J.J., Walsh C., Gorey S., Harris K., Hervella P., Iglesias-Rey R., Jern C., Li L., Miyamoto N., Montaner J. (2023). C-Reactive Protein, Interleukin-6, and Vascular Recurrence After Stroke: An Individual Participant Data Meta-Analysis. Stroke.

[8] di Gioia C.R.T., Ascione A., Carletti R., Giordano C. (2023). Thoracic Aorta: Anatomy and Pathology. Diagnostics.

[9] Nilsson P.M., Khalili P., Franklin S.S. (2014). Blood pressure and pulse wave velocity as metrics for evaluating pathologic ageing of the cardiovascular system. Blood Press..

[10] Ohyama Y., Ambale-Venkatesh B., Noda C., Chugh A.R., Teixido-Tura G., Kim J.-Y., Donekal S., Yoneyama K., Gjesdal O., Redheuil A. (2018). Association of Aortic Stiffness With Left Ventricular Remodeling and Reduced Left Ventricular Function Measured by Magnetic Resonance Imaging. Circ. Cardiovasc. Imaging.

[11] Herment A., Kachenoura N., Lefort M., Bensalah M., Dogui A., Frouin F., Mousseaux E., Cesare A.D. (2010). Automated segmentation of the aorta from phase contrast MR images: Validation against expert tracing in healthy volunteers and in patients with a dilated aorta. J. Magn. Reason. Imaging.

[12] Herment A., Lefort M., Kachenoura N., Cesare A.D., Taviani V., Graves M.J., Pellot-Barakat C., Frouin F., Mousseaux E. (2011). Automated estimation of aortic strain from steady-state free-precession and phase contrast MR images. Magn. Reason. Med..

[13] Redheuil A., Yu W.-C., Wu C.O., Mousseaux E., de Cesare A., Yan R., Kachenoura N., Bluemke D., Lima J.A.C. (2010). Reduced Ascending Aortic Strain and Distensibility. Hypertension.

[14] Nettleton J.A., Steffen L.M., Mayer-Davis E.J., Jenny N.S., Jiang R., Herrington D.M., Jacobs D.R. (2006). Dietary patterns are associated with biochemical markers of inflammation and endothelial activation in the Multi-Ethnic Study of Atherosclerosis (MESA). Am. J. Clin. Nutr..

[15] Lutsey P.L., Cushman M., Steffen L.M., Green D., Barr R.G., Herrington D., Ouyang P., Folsom A.R. (2006). Plasma hemostatic factors and endothelial markers in four racial/ethnic groups: The MESA study. J. Thromb. Haemost..

[16] Cushman M., Cornell E.S., Howard P.R., Bovill E.G., Tracy R.P. (1995). Laboratory methods and quality assurance in the Cardiovascular Health Study. Clin. Chem..

[17] Jain S., Khera R., Corrales–Medina V.F., Townsend R.R., Chirinos J.A. (2014). Inflammation and arterial stiffness in humans. Atherosclerosis.

[18] Johansen N.B., Vistisen D., Brunner E.J., Tabák A.G., Shipley M.J., Wilkinson I.B., McEniery C.M., Roden M., Herder C., Kivimäki M. (2012). Determinants of Aortic Stiffness: 16-Year Follow-Up of the Whitehall II Study. PLoS ONE.

[19] McEniery C.M., Spratt M., Munnery M., Yarnell J., Lowe G.D., Rumley A., Gallacher J., Ben-Shlomo Y., Cockcroft J.R., Wilkinson I.B. (2010). An Analysis of Prospective Risk Factors for Aortic Stiffness in Men. Hypertension.

[20] van Bussel B.C., Schouten F., Henry R.M., Schalkwijk C.G., de Boer M.R., Ferreira I., Smulders Y.M., Twisk J.W., Stehouwer C.D. (2011). Endothelial Dysfunction and Low-Grade Inflammation Are Associated With Greater Arterial Stiffness Over a 6-Year Period. Hypertension.

[21] Jae S.Y., Heffernan K.S., Yoon E.S., Park S.H., Choi Y.-H., Fernhall B., Park J.B. (2012). Pulsatile Stress, Inflammation and Change in Arterial Stiffness. J. Atheroscler. Thromb..

[22] Tomiyama H., Hashimoto H., Tanaka H., Matsumoto C., Odaira M., Yamada J., Yoshida M., Shiina K., Nagata M., Yamashina A. (2010). Continuous Smoking and Progression of Arterial Stiffening A Prospective Study. J. Am. Coll. Cardiol..

[23] Jabati S., Fareed J., Liles J., Otto A., Hoppensteadt D., Bontekoe J., Phan T., Walborn A., Syed M. (2018). Biomarkers of Inflammation, Thrombogenesis, and Collagen Turnover in Patients With Atrial Fibrillation. Clin. Appl. Thromb./Hemost..

[24] Ridker P.M. (2016). From C-Reactive Protein to Interleukin-6 to Interleukin-1. Circ. Res..

[25] Wirtz D.C., Heller K.-D., Miltner O., Zilkens K.-W., Wolff J.M. (2000). Interleukin-6, a potential inflammatory marker after total joint replacement. Int. Orthop..

[26] Sesso H.D., Wang L., Buring J.E., Ridker P.M., Gaziano J.M. (2007). Comparison of Interleukin-6 and C-Reactive Protein for the Risk of Developing Hypertension in Women. Hypertension.

[27] Ridker P.M., Everett B.M., Thuren T., MacFadyen J.G., Chang W.H., Ballantyne C., Fonseca F., Nicolau J., Koenig W., Anker S.D. (2017). Antiinflammatory Therapy with Canakinumab for Atherosclerotic Disease. N. Engl. J. Med..

[28] Redheuil A., Yu W.-C., Mousseaux E., Harouni A.A., Kachenoura N., Wu C.O., Bluemke D., Lima J.A.C. (2011). Age-Related Changes in Aortic Arch Geometry Relationship With Proximal Aortic Function and Left Ventricular Mass and Remodeling. J. Am. Coll. Cardiol..

[29] Nwabuo C.C., Moreira H.T., Vasconcellos H.D., Ambale-Venkatesh B., Yoneyama K., Ohyama Y., Sharma R.K., Armstrong A.C., Ostovaneh M.R., Lewis C.E. (2017). Association of Aortic Root Dilation from Early Adulthood to Middle Age with Cardiac Structure and Function: The CARDIA Study. J. Am. Soc. Echocardiog..

[30] Ohyama Y., Redheuil A., Kachenoura N., Venkatesh B.A., Lima J.A.C. (2018). Imaging Insights on the Aorta in Aging. Circ. Cardiovasc. Imaging.

